# An Embeddable Algorithm for Automatic Garbage Detection Based on Complex Marine Environment

**DOI:** 10.3390/s21196391

**Published:** 2021-09-24

**Authors:** Hongjie Deng, Daji Ergu, Fangyao Liu, Bo Ma, Ying Cai

**Affiliations:** Key Laboratory of Electronic and Information Engineering, Southwest Minzu University, State Ethnic Affairs Commission, Chengdu 610041, China; denghj1221@163.com (H.D.); FLIU028@163.com (F.L.); martbox@163.com (B.M.); 21500121@swun.edu.cn (Y.C.)

**Keywords:** deep learning, object detection, instance segmentation, marine ecology, the attentional mechanism, dilated convolution

## Abstract

With the continuous development of artificial intelligence, embedding object detection algorithms into autonomous underwater detectors for marine garbage cleanup has become an emerging application area. Considering the complexity of the marine environment and the low resolution of the images taken by underwater detectors, this paper proposes an improved algorithm based on Mask R-CNN, with the aim of achieving high accuracy marine garbage detection and instance segmentation. First, the idea of dilated convolution is introduced in the Feature Pyramid Network to enhance feature extraction ability for small objects. Secondly, the spatial-channel attention mechanism is used to make features learn adaptively. It can effectively focus attention on detection objects. Third, the re-scoring branch is added to improve the accuracy of instance segmentation by scoring the predicted masks based on the method of Generalized Intersection over Union. Finally, we train the proposed algorithm in this paper on the Transcan dataset, evaluating its effectiveness by various metrics and comparing it with existing algorithms. The experimental results show that compared to the baseline provided by the Transcan dataset, the algorithm in this paper improves the mAP indexes on the two tasks of garbage detection and instance segmentation by 9.6 and 5.0, respectively, which significantly improves the algorithm performance. Thus, it can be better applied in the marine environment and achieve high precision object detection and instance segmentation.

## 1. Introduction

The marine ecosystem is the essential condition for the survival and development of marine organisms. Therefore, when the amount of external environmental changes exceeds the tolerance limit of the biological community, it will directly affect the virtuous cycle of the ecosystem, thus destroying the ecosystem.

In human development, manufactured or processed solid waste is inevitably discarded into the ocean, and this category of waste is called marine litter. To some extent, it affects the marine landscape, threatens the safety of shipping routes, and impacts the health of the marine ecosystem, which in turn hurts the marine economy. In addition, when the total amount of marine litter exceeds the tolerance limit of marine biological communities, it will directly affect the virtuous cycle of the marine ecosystem, leading to the deterioration of the ecological environment, putting biological resources under threat and eventually causing damage to the marine life ecological environment. In order to address the root cause of the problem, it is necessary to prevent the occurrence of litter and microplastic discharges into the ocean in the long term. Therefore, a correct understanding of the types of marine litter and litter cleanup can help reduce the impact of marine litter on the marine ecological environment. This carries an important practical significance for future marine ecological environment management and sustainable development [1]. Based on protecting the marine ecosystem, various methods of marine litter cleanup have been proposed and adopted by different environmental departments within government agencies, but they are not widely used. With the development of deep learning, computer vision offers a viable solution for this class of tasks [2], which are achieved by applying autonomous underwater detectors fitted with object detection algorithms. It can detect and locate the garbage in the seabed, thus making automatic or manned garbage cleanup possible. Thus, the performance of the target detection algorithm directly determines the completion of this task.

Nowadays, object detection algorithms can be divided into two types, namely one-stage and two-stage.

The one-stage algorithm directly regresses the class and coordinates of objects, and its representative algorithms are mainly the Yolo series and SSD [3], etc. The core idea of the Yolo family of algorithms is to use the entire graph as the input to the network, thus directly outputting the outer boxes and categories of the target. Yolov1 [4] divided the whole map into S × S grids and made individual predictions for the object in the grid where the center point is located. Both Yolov2 [5] and SSD use a priori frames, with each grid pre-set with a set of borders of different sizes and aspect ratios to cover the whole image at different locations and multiple scales; the former extracts the scale information of the a priori frames by clustering to improve the detection accuracy, and the latter uses a multi-scale feature map for object prediction. Yolov3 [6] introduced the Darknet-53 network to extract features and use multi-scale features for object detection. Yolov4 [7] retained the head part of Yolov3 and modified the backbone network to CSPDarknet53, while the idea of SPP [8] (spatial pyramid pooling) was used to expand the receptive field. The one-stage algorithm is simple in structure and has good real-time performance in detection, but the detection accuracy is poor compared to the two-stage algorithm.

The two-stage algorithm divides the detection task into two stages: candidate region generation, classification of the candidate regions, and border regression. The R-CNN family of algorithms is a classic representative of this type. R-CNN [9] borrows the idea of Selective Search [10], which extracts 2000 candidate regions on the input image, extracts feature from them, and then uses an SVM classifier to complete the classification. Fast R-CNN [11] feeds the whole image into the CNN for feature extraction and generates a fixed-size feature map for subsequent detection by RoI Pooling. Faster R-CNN [12] enhances the semantic and spatial information of features by Feature Pyramid Network (FPN) [13] and introduces RPN module to generate candidate regions based on a multi-scale anchor box. Finally, Mask R-CNN [14] adds Mask branch to the former, on which the feature map is classified pixel-by-pixel by Fully Convolution Network (FCN) [15], thus realizing instance segmentation under object detection.

In the marine environment, there is not only garbage that we need to detect, but also some objects that will have an impact on our detection. Furthermore, the image resolution in the dataset we use is low. All of the above factors can lead to ineffective feature extraction in the network training, thus making the obtained feature information weak. To achieve the task of marine garbage detection efficiently, this paper introduces ideas such as dilated convolution [16] and attention mechanism [17] to improve the basic structure of Mask R-CNN. Then the re-scoring branch is added. Finally, we design an object detection and instance segmentation model to achieve optimal performance in this scenario. All these improvements were made so that the algorithm could be embedded in the underwater detector for efficient garbage cleaning.

The contribution of this paper is as follows:

- Add dilated convolution to the FPN structure. The purpose of this is to enhance feature extraction for small objects.
- Introduce a spatial attention mechanism and channel attention mechanism. The attention mapping is multiplied into the input feature mapping for adaptive feature refinement, thus enabling adaptive learning of features and increasing the weights of the key features.
- Add the re-scoring branch. The overlap between the real mask and the predicted mask is calculated and scored by Generalized Intersection over Union (GIOU) [18], and the scores are subsequently multiplied with the classification scores as the confidence level of the mask; finally, the effect of instance segmentation is made more accurate by network training.

## 2. Related Works

### 2.1. Problems Exist in Task Realization

There are two key facets of the task to be implemented in this paper: One concerns how to extract the object features efficiently, and the other concerns how to perform accurate detection and instance segmentation of the object. This can be difficult as there are multiple organisms in the ocean, and the environment is complex. Moreover, the images taken by vision-equipped autonomous underwater vehicles have low resolution. These problems inhibit to some extent the effectiveness of the algorithms exhibited in this application scenario. In other words, this paper focuses on how to perform efficient feature extraction and focus on detection objects in low-resolution images to improve the effectiveness of target detection and instance segmentation.

### 2.2. Existing Work

Although achieving litter cleanup in the marine environment is a worthwhile topic of inquiry for both the environmental protection field and industry, there are relatively few studies on the detection and instance segmentation of marine litter by deep learning at this stage. Valdenegro-Toro M [19] proposes using Autonomous Underwater Vehicles to detect submerged marine debris from Forward-Looking Sonar (FLS) imagery, train a Convolutional Neural Network to classify the images, and use this classifier as an object detector in a sliding window fashion. Similar to this application scenario, Kylili et al. [20] proposed a method that can automatically identify floating marine plastics based on deep learning. Tharani et al. [21] consider that some of the popular object detectors fail to identify smaller objects present, so they employ an attention layer to enforce the algorithm to focus on smaller objects. Michael et al. [22] think that autonomous underwater vehicles (AUVs) could very well contribute to protecting the marine ecological environment by finding and eventually removing trash. Thus, they train some convolutional neural network structures for object detection on a large and publicly available dataset of actual debris in open-water locations and evaluate their detection effectiveness to provide insights for developing detection capabilities for AUVs to remove underwater trash. Jun-Ichiro et al. [23] use YOLO v3 to detect underwater sea life and debris floating on the ocean surface, thus arguing for the feasibility of using autonomous robots, including commercial UAVs and AUVs, to monitor the marine environment.

Since the marine environment is complex, and most algorithms are not applicable in this scenario, many experimental results have been relatively poor. To solve this problem within the task of object detection, Tan [24] proposes using a deep proposal mechanism (DPM), which can achieve a high-performance, complex object detection. Chen et al. [25] propose a novel module called an adaptive convolution block (ACB). Due to such adaptive convolution, the enhanced features can pay more attention to the relevant objects, suppress the interference information caused by irrelevant surroundings, and efficiently improve the detection accuracy. Sun et al. [26] propose a unified part-based convolutional neural network (PBNet). It treats a composite object as a group of parts and incorporates part information into context information to improve composite object detection. Sun et al. [27] constructed a Mask-SSD network, increasing the SSD performance for detecting target objects of small size by enhancing detection features with contextual information and introducing a segmentation mask to eliminate background regions. Wu et al. [28] have proposed a new pipeline for salient end-to-end instance segmentation (SIS) that predicts a class-agnostic mask for each detected salient instance. Tian et al. [29] applied the U-Net backbone to Mask scoring R-CNN. By improving the processing ability of image boundary pixels, the effect of instance segmentation is optimized.

Therefore, combining the ideas of the above-mentioned improvement methods, this paper focuses on the three aspects of object attention, feature extraction ability, and instance segmentation effect as the key research content. First, based on the original structure of Mask R-CNN, the addition of a spatial-channel attention mechanism enhances the network′s focus on object features. Then, the idea of dilated convolution is applied to the lateral connection of FPN. It is used to improve the feature extraction capability for low-resolution images in the dataset. Finally, the re-scoring branch re-evaluates the generated mask effect to improve the effect of instance segmentation.

## 3. Mask R-CNN

Mask R-CNN is a method proposed by Kaiming et al. that can be used for object detection and instance segmentation.

**Figure 1:**
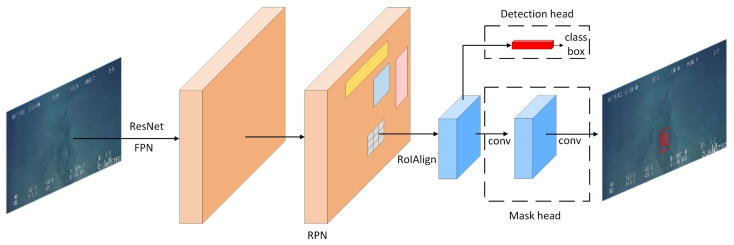
The structure of Mask R-CNN.

The implementation flow is shown in Figure 1. The algorithm is composed of two parts: the detection part and the instance segmentation part.

Mask R-CNN is based on Faster R-CNN and combines ResNet [30] with FPN as a feature extraction network to obtain the feature map by multi-layer convolutional feature extraction of the image. Then, the Region Proposal Network (RPN) is used to generate proposal of regions for the object. Finally, object detection and instance segmentation are achieved by detection head and mask head.

However, in the complex ocean scene, there are some shortcomings in using Mask R-CNN directly due to several external factors such as many distracting objects and low resolution of captured images. The main problems are difficulty extracting object features and the low attention to the objects for detection. Therefore, in order to achieve high-precision marine litter detection and instance segmentation, this paper optimizes and improves the model based on Mask R-CNN.

## 4. Methods

Due to the relative complexity of the application scenarios studied in this paper, conventional network models present a challenge to achieving high results. Therefore, this paper improves on the Mask R-CNN. First, it does this by adding dilated convolution to the FPN to capture multi-scale feature information and, then, by using the spatial-channel attention mechanism to increase the attention of the object. Finally, the re-scoring branch is introduced to re-evaluate the generated masks based on GIOU.

### 4.1. Lateral Connection of FPN Based on Dilated Convolution

When the features are extracted through the backbone network, the low-level feature maps contain less semantic information but rich location information. The opposite is true for the high-level feature maps. The feature pyramid structure is used in Mask R-CNN, which is a multi-scale feature extraction method. It is composed of three parts:

- Bottom-up: extracting feature information
- Top-down: recovering the original dimensions of each level by up-sampling the feature map
- Lateral connection: adding two feature maps with the same scale. Improving both the semantic and spatial information of the feature maps

**Figure 2:**
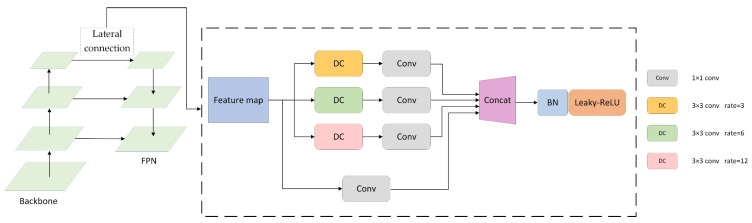
Improvements based on lateral connections in Feature Pyramid Network.

Mask R-CNN introduces FPN to extract and optimize the features of the image. As the depth of the network increases, although this operation increases the receptive field of each element on the feature map, allowing them to observe a larger range of the input image as a way to improve the semantic information contained in itself. However, for low-resolution images, the image size is already small, and after the convolution operation, the obtained feature map becomes even smaller, which greatly reduces the spatial information contained in the feature map; thus, this will seriously affect the object localization and instance segmentation. Therefore, in order not to reduce the spatial resolution of the feature map during the convolution process and still expand the receptive field, we introduce the dilated convolution. In addition, the dilated convolution can change the receptive field size by setting a different dilation rate, which helps the network to obtain the multi-scale information of the object so that the semantic and spatial information of the object can be optimized by the multi-scale information during the network training, thus improving the accuracy of the object detection and instance segmentation. Figure 2 shows the improved FPN structure.

Unlike the traditional FPN, which only uses one 1 × 1 convolution in the lateral connection, the improved network adds three dilated convolutions, i.e., 3 × 3 conv with sampling rates of 6, 12, and 18, to sample the feature maps. The structure is designed to capture the feature information of the image at multiple scales. Then, the feature map is downscaled by 1 × 1 conv and concatenated with the original feature map to obtain more image features. Finally, the channel number of the feature map is decreased to our desired value by using 1 × 1 conv, and the results are input sequentially into the BN layer with the Leaky-ReLU function in order. In this way, we can achieve the feature fusion in the lateral connection in FPN. This operation enhances the ability to extract features from low-resolution images while ensuring no problems such as gradient explosion or disappearance. Experiments show that using this lateral connection structure based on dilated convolution makes image features easier to extract. This is used to achieve more accurate target localization and classification, which results in a better instance segmentation effect.

### 4.2. Optimization of Feature Extraction Based on Spatial-Channel Attention Mechanism

**Figure 3:**
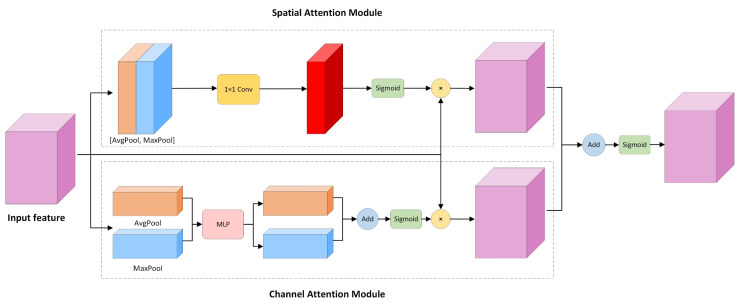
A Spatial-channel attention mechanism module that can be embedded in a convolutional neural network.

Traditional object detection and instance segmentation algorithms only extract features from images by convolutional neural networks. In other words, the same attention is given to each pixel in the image and eventually the network is trained to achieve the corresponding task. However, this may lead to an increase in the training time and an extraction of too many useless features. In particular, in the application scenario of this paper, a series of influencing factors such as the large number of objects in the marine environment and the low resolution of the captured images all lead to failure of the network to focus on the research object. Therefore, we designed a spatial-channel attention module to solve this problem, which is added to each block of ResNeXt [31]. By continually training the network, it is able to automatically determine how much attention should be assigned to each part of the input. Based on this operation, the attention given to the object area is increased, while the attention of the influenced object is reduced thus providing accurate attention to the features extracted by the convolutional neural network. With this module, the object features are given higher weights and enhanced extraction of the object features, thereby optimizing the training time and improving the model’s accuracy. The structure is shown in Figure 3. The structure contains two modules, namely the spatial attention module and the channel attention module.

First, we generated the corresponding feature maps (H × W × C) by feature extraction of the images and sent these to the spatial attention module and the channel attention module shown in Figure 3. The channel attention module mainly performs both global maximum pooling and global average pooling operations on the width and height dimensions of the feature map. It generates two feature maps with size of 1 × 1 after this operation. The number of channels of the feature map is C. Subsequently, the feature maps are fed separately into the MLP, which is composed of a two-layer neural network with shared parameters in the network. Then, the feature maps after MLP are summed up via element-wise to generate the channel attention feature, M_c. Finally, it is multiplied with the input feature map to obtain the feature map containing the channel information.

Similarly, the spatial attention module performs channel-based global max pooling and global average pooling on the input feature maps. The two H × W × 1 feature maps are obtained, and then, the two feature maps are concatenated based on channels, and the channel number of the feature maps is reduced to 1 using 7 × 7 convolution, i.e., H × W × 1. The same sigmoid function generates the spatial attention feature, i.e., M_s, and multiplies it element-wise with the input feature map to obtain the feature map with spatial information. These add up the final feature maps from the two modules. Finally, the channel number of the feature map is reduced by 1 × 1 convolution. Thus, feature fusion based on both spatial and channel dimensions is achieved, providing more valuable features for subsequent network modules.

### 4.3. Re-Scoring Brach

In the Mask R-CNN model, mask generation is implemented by mask head. It uses RoIAlign to process the feature maps and implements pixel-by-pixel classification through a fully convolutional neural network to generate the mask corresponding to the object. In the instance segmentation, the output mask score is shared with the bounding box and calculated for the object region’s classification confidence. But this score does not necessarily represent the actual quality and completeness of each object’s mask. Even if each object gets a better score for bounding box localization and classification, which results in a higher mask score, it is possible that the actual quality of the mask is poor. Therefore, scoring Mask using such a classification score does not necessarily demonstrate the effect of instance segmentation in a realistic way, thus reducing the evaluation results. Besides, it is more difficult to achieve instance segmentation of the object in low-resolution images.

**Figure 4:**
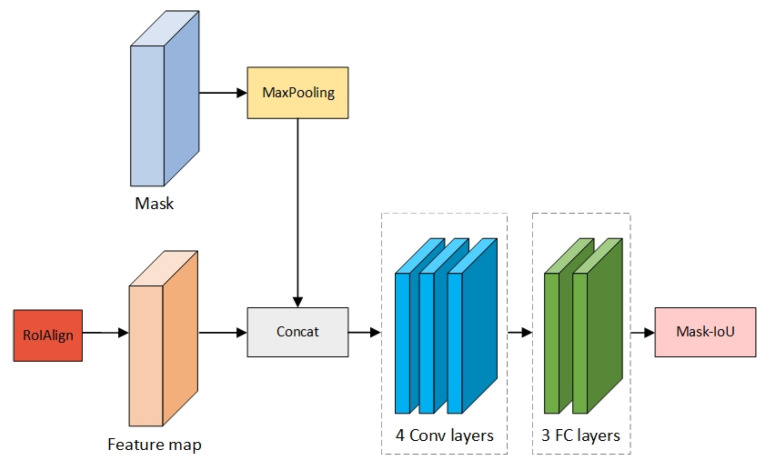
The structure of Re-scoring branch.

To solve this problem, we divide the score of the mask into the classification score and the score of Intersection over Union (IOU). Through the training of the network, the IOU between the ground truth and the predicted mask is calculated, as well as being continuously optimized to improve the accuracy of the instance segmentation. Since the classification score can be calculated from the R-CNN head, the re-scoring branch is introduced. The network structure is shown in Figure 4.

We connect the feature of the RoIAlign layer with the predicted mask as the input of the re-scoring branch. It measures the accuracy of the segmentation by calculating the pixel-level IOU values between the predicted mask obtained from the mask branch and the mask corresponding to the ground truth. In other words, the task of re-scoring is to regress the predicted Mask IOU so that the predicted Mask IOU is infinitely close to the Mask IOU of Ground Truth (GT) obtained via the Mask branch. The predicted Mask IOU represents the value of Mask IOU predicted by the re-scoring branch. The GT Mask IOU represents the value of IOU calculated from the Mask branch predicted by the mask and the real mask. Thus, the score of masks can be expressed by the equation:

(1) $$S_{\mathrm{mask}}=S_{\mathrm{cls}}\cdot S_{\mathrm{GIOU}}$$

**Figure 5:**
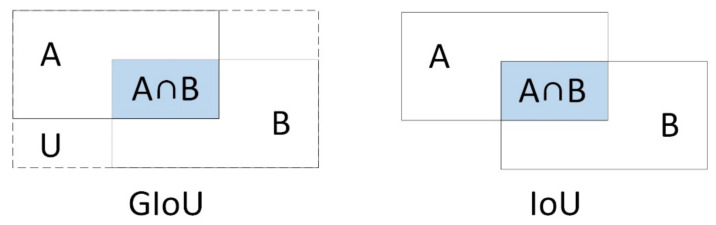
Comparison of GIOU and IOU.

Compared to IOU, GIOU is able to optimize the non-overlapping part, which is the reason why we use GIOU in this paper when computing mask IOU. Figure 5 represents the schematic diagrams of the IOU and GIOU, respectively.

It can be seen that GIOU is improved based on IOU. The representation of both is as follows:

(2) $$\mathrm{IOU}=\frac{|A\cap B|}{|A\cup B|}$$

(3) $$\mathrm{GIOU}=\mathrm{IOU}-\frac{A^{c}-U}{A^{c}}$$

A and B represent the regions of the predicted mask and GT mask, respectively. $A^{c}$ and U are the intersection and union of the above two. Since GIOU introduces the concept of $A^{c}$, the regression can still be optimized when the two regions do not overlap, better reflecting the overlap of the two masks. In summary, it retains the original characteristics of IOU while weakening its drawbacks. The final GIOU loss function is:

(4) $$L_{\mathrm{GIOU}}=1-\mathrm{GIOU}$$

### 4.4. Improved Network Model Structure

**Figure 6:**
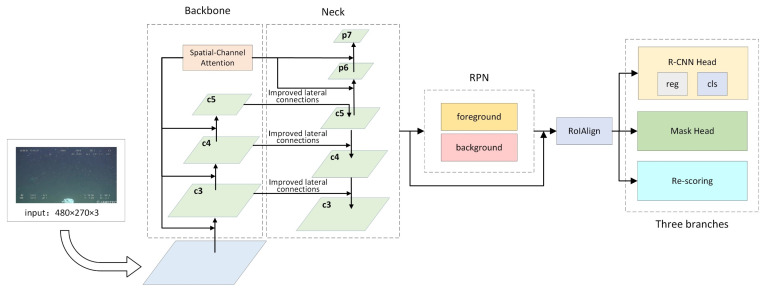
The overall structure of the proposed method in this paper.

In this paper, the proposed method is shown in Figure 6.

We have improved three main modules: the backbone network, the neck network, and the head. First, features of the images are extracted by ResNeXt-101, and then, the spatial-channel attention mechanism is added to the backbone network to increase the attention to object features. The low resolution of the images in the dataset results in poor detection of small objects. To solve this problem, we introduce a feature extraction and optimization module in the lateral connection of FPN. This improves the receptive field of the feature map by the idea of dilated convolution. Eventually, the re-scoring branch is added to the original structure of Mask R-CNN, and the mask score is re-evaluated by calculating and regressing the IOU values between the generated mask and GT mask to achieve a more accurate instance segmentation.

## 5. Experiments

To verify that the improved network model in this paper has significant advantages over other models for the task of marine garbage detection and instance segmentation, we conducted several experiments to illustrate the effectiveness of the model.

### 5.1. Datasets

**Figure 7:**
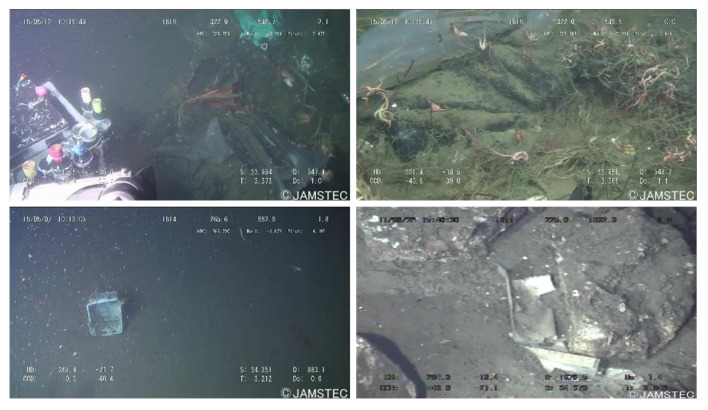
Display of some images in the Trashcan dataset.

In this paper, we used the Transhcan dataset [32], which is composed of underwater garbage images collected from various sources and which contains a total of 7212 images, and classified it into 22 categories. The image format in this dataset is jpg, with a resolution of 480 × 270. The dataset is annotated in two forms: bounding box and segmented labels. The aim is to develop efficient and accurate methods for garbage detection and instance segmentation. Figure 7 shows some of the images in the dataset. To improve the generalization ability and robustness exhibited by the model after training, we use various data enhancement techniques, such as Crop and Flips.

### 5.2. Experimental Platform and Parameters

The configuration parameters of the experimental platform implemented by the algorithm in this paper are shown in Table 1.

### 5.3. Evaluation Metrics

In the tasks implemented in this paper, precision and recall are often used as metrics to validate the model accuracy:

(5) $$\mathrm{Precision}=\frac{\mathrm{TP}}{\mathrm{TP}+\mathrm{FP}}$$

(6) $$\mathrm{Recall}=\frac{\mathrm{TP}}{\mathrm{TP}+\mathrm{FN}}$$

Here, TP (True-Positive) means that the actual category of the test object is the same as the predicted result, i.e., all positive; FP (False-Positive) means that the test object is predicted to be positive but is negative; FN (False-Negative) refers to a situation where the test object is predicted to be negative but is actually positive. To describe the effect presented by the model in this paper, we use AP (Average Precision) and mAP (mean Average Precision) to evaluate the accuracy of the model. The AP value is the mean value of the precision value under different recalls, i.e., the size of the area under the PR curve, and mAP value is the average of the mean precision for all object classes. The formula is as follows:

(7) $$\mathrm{AP}=\int_{0}^{1}P\left(R\right)\mathrm{dR}$$

(8) $$\mathrm{mAP}=\frac{\sum_{i=1}^{N}{\mathrm{AP}}_{i}}{N}$$

where P, R, and N represent precision, recall, and the total number of instances in all categories, respectively. For mAP, we also used the five metrics including mAP_50_, mAP_75_, mAP_s_, mAP_m_, and mAP_l_. Among them, mAP_50_ and mAP_75_ represent mAP with confidence levels of 0.5 and 0.75, respectively, while mAP_s_, mAP_m_, and mAP_l_ represent mAP with object areas less than 32^2^, between 32^2^ and 96^2^, and larger than 96^2^, respectively.

### 5.4. Training

**Figure 8:**
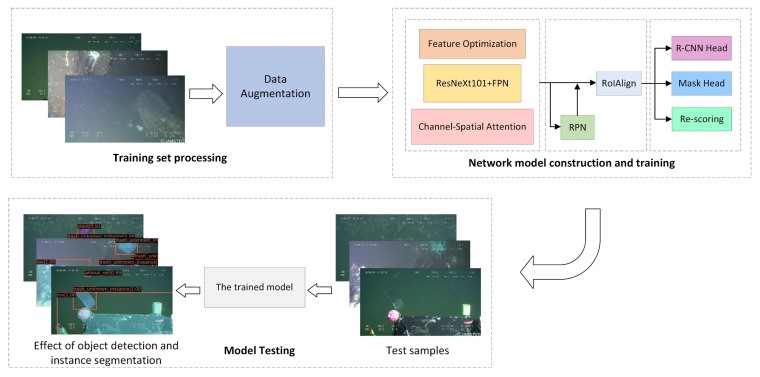
Experimental procedure.

The experiment flow of this paper is shown in Figure 8.

Firstly, a series of data enhancement techniques, such as image rotation and cropping, are applied to the original dataset to reduce the impact of low image resolution and ensure that the image features can be extracted effectively afterward. Subsequently, the processed test dataset is fed into the optimized neural network in this paper for training.

In the training phase, all models were trained for 100 epochs. The batch size is set to 12, and the initial learning rate is 0.01. Network parameters are also optimized using stochastic gradient descent (SGD). The momentum, as well as decay weights, are 0.9 and 0.0001, respectively.

### 5.5. Results of the Experiment

#### 5.5.1. Visualization of Test Images

**Figure 9:**
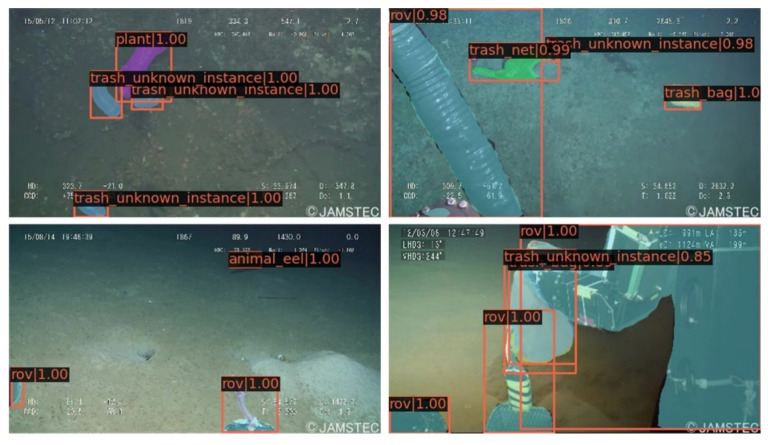
Visualization of our model in performance testing.

Figure 9 illustrates the effectiveness of the improved network in this paper on the task of marine garbage detection and instance segmentation. It can be seen that the network can achieve accurate object localization and classification. At the same time, the effect of mask generation corresponding to the object is also better.

#### 5.5.2. Performance Comparison of Different Algorithms

To scientifically demonstrate the performance of our method on the two tasks of target detection and instance segmentation, we trained and tested the proposed method simultaneously with multiple methods on the same dataset and compared the test results. Table 2 and Table 3 show the performance of each algorithm on detection and instance segmentation.

As shown in Table 2 and Table 3, the method proposed in this paper achieves better results when implementing the above two tasks. For the task of object detection, the mAP of this model increased by 9.6%, 4.7%, and 2.4% compared with the three algorithms i.e., Faster R-CNN, RetinaNet [33], and FCOS [34], respectively. It can be seen that the comprehensive performance of the method proposed in this paper is superior to these algorithms. In addition, the test effect has been greatly optimized in different size of area. On instance segmentation, the method in this paper improves 5.0%, 4.2%, and 3.9% on mAP compared to Mask R-CNN, SOLO [35] and CondInst [36], respectively. Compared with CondInst, which has been proposed recently, the improvement in mAPs, mAPm, and mAPl is 1.6, 1.4, and 5.6, respectively. This indicates that this method can be better applied to the task of instance segmentation in this scenario compared to other algorithms. In general, the method proposed in this paper achieves good results in object detection and instance segmentation tasks, and the performance is greatly improved compared to Mask R-CNN.

To verify the effectiveness of the improved functional module based on Mask R-CNN in this paper, we use Mask R-CNN as the benchmark algorithm. While keeping the original structure unchanged, the improved modules are replaced one by one, and the overall effect is evaluated by training and testing the network.

Table 4 shows the degree of effect optimization brought about by our improved method for the object detection task. Compared with Mask R-CNN, the improved lateral connection resulted in an improvement of 6.9, 9.5, and 6.7 for mAP, mAP50, and mAP75, respectively. Similarly, using the spatial-channel attention mechanism improved mAP, mAP50, and mAP75 by 7.4, 9.2, and 8.3, respectively. However, it can be seen from the table that for objects of different area sizes, the improved lateral connection has the most significant improvement in the detection of small objects. mAPs improved by 7.7. At the same time, the spatial-channel attention mechanism only made it improve by 6.2. However, the enhancement effect is similar in all other aspects. Therefore, our proposed method has a great advantage over Mask R-CNN. Combining these two modules can achieve optimized object detection effects in complex environments, especially small target detection.

Table 5 shows the effect that the application of the re-scoring branch improves on the instance segmentation task compared to Mask R-CNN. It can be seen that a large improvement is achieved both in the overall effect and in the effect of instance segmentation for objects of different sizes. Thus, this shows that the re-scoring branch is able to optimize the generation of the mask, which substantially improves the accuracy of the instance segmentation.

## 6. Conclusions and Outlooks

At this stage, with the development of deep learning, the field of marine science is also gradually using this emerging technology. Especially in this application of marine environmental monitoring, numerous scholars have used machine learning and neural network approaches to automatically detect seabed litter [37,38,39].

Based on the previous research, we have improved Mask R-CNN. First, spatial-channel attention is introduced in feature extraction to improve the attention of object features. Second, the idea of dilated convolution is applied to FPN, which optimizes feature information. Finally, the re-scoring branch is added to the original structure of Mask R-CNN to improve the effect of instance segmentation

The experimental results show that the algorithm proposed in this paper has a better performance in object detection and instance segmentation compared with Mask R-CNN. This enables the model to be deployed on autonomous underwater detectors for automatic garbage detection. Thus, subsequent marine debris removal tasks are realized. Furthermore, this algorithm can be extended to other object detection and instance segmentation tasks that are easily affected by the environment (for example, crop detection, etc.).

However, in the current work, there are still some problems. For example, the number of parameters in the network is large and cannot be effectively detected in real time. Therefore, in future work, we will consider focusing on this aspect to reduce the number of parameters by optimizing the network structure to make it a lightweight network, thus enhancing the real-time implementation of related tasks.

## Figures and Tables

**Table 1:** Configuration parameters.

Device	Configuration
Operating system	Ubuntu18.04
Processor	Intel(R) Xeon(R)Silver 4110 CPU @2.10GHz
GPU	GeForce RTX 2080Ti
GPU accelerator	CUDA 10.2
Frame	Pytorch
Compilers	Pycharm
Scripting language	Python3.7

**Table 2:** The comparisons of numerous detection methods.

Method	mAP	mAP_50_	mAP_75_	mAP_s_	mAP_m_	mAP_l_
Faster R-CNN	34.5	55.4	38.1	27.6	36.2	51.4
RetinaNet	39.4	57.3	44.7	31.1	42.9	58.5
FCOS	41.7	60.4	45.9	33.2	44.6	60.2
Our method	44.1	65.0	48.1	35.4	48.3	65.7

**Table 3:** The comparisons of numerous instance segmentation methods.

Method	mAP	mAP_50_	mAP_75_	mAP_s_	mAP_m_	mAP_l_
Mask R-CNN	30.9	56.7	29.5	26.3	36.1	55.0
SOLO	31.7	55.9	31.4	28.1	37.3	57.2
CondInst	32.0	57.1	31.3	27.8	37.6	55.7
Our method	35.9	60.2	36.1	29.4	39.0	61.3

**Table 4:** Using different methods for object detection.

Method	mAP	mAP_50_	mAP_75_	mAP_s_	mAP_m_	mAP_l_
Mask R-CNN	36.2	55.7	39.3	27.9	38.4	52.6
Mask R-CNN+ Improved lateral connection	43.1	65.2	46.0	35.6	48.3	65.2
Mask R-CNN+ The spatial-channel attention mechanism	43.6	64.9	47.6	34.1	48.0	64.5

**Table 5:** Using different methods for instance segmentation.

Method	mAP	mAP_50_	mAP_75_	mAP_s_	mAP_m_	mAP_l_
Mask R-CNN	30.9	56.7	29.5	26.3	36.1	55.0
Mask R-CNN+ re-scoring	35.1	59.2	35.8	28.5	38.6	59.8

## Data Availability

The data presented in this study are available on request from the first author.
